# Combined Application of Manure and Chemical Fertilizers Alters Soil Environmental Variables and Improves Soil Fungal Community Composition and Rice Grain Yield

**DOI:** 10.3389/fmicb.2022.856355

**Published:** 2022-07-14

**Authors:** Anas Iqbal, Izhar Ali, Pengli Yuan, Rayyan Khan, He Liang, Shanqing Wei, Ligeng Jiang

**Affiliations:** ^1^College of Life Science and Technology, Guangxi University, Nanning, China; ^2^College of Agriculture, Guangxi University, Nanning, China; ^3^Key Laboratory of Tobacco Biology and Processing, Ministry of Agriculture and Rural Affairs, Tobacco Research Institute, Chinese Academy of Agricultural Sciences, Qingdao, China

**Keywords:** chemical fertilizer, fungal community, manure, rice, soil properties

## Abstract

Soil microorganisms play vital roles in energy flow and soil nutrient cycling and, thus, are important for crop production. A detailed understanding of the complex responses of microbial communities to diverse organic manure and chemical fertilizers (CFs) is crucial for agroecosystem sustainability. However, little is known about the response of soil fungal communities and soil nutrients to manure and CFs, especially under double-rice cropping systems. In this study, we investigated the effects of the application of combined manure and CFs to various fertilization strategies, such as no N fertilizer (Neg-CF); 100% chemical fertilizer (Pos-CF); 60% cattle manure (CM) + 40% CF (high-CM); 30% CM + 70% CF (low-CM); 60% poultry manure (PM) + 40% CF (high-PM), and 30% PM + 70% CF (low-PM) on soil fungal communities' structure and diversity, soil environmental variables, and rice yield. Results showed that synthetic fertilizer plus manure addition significantly increased the soil fertility and rice grain yield compared to sole CFs' application. Moreover, the addition of manure significantly changed the soil fungal community structure and increased the relative abundance of fungi such as phyla Ascomycota, Basidiomycota, Mortierellomycota, and Rozellomycota. The relative abundances dramatically differed at each taxonomic level, especially between manured and non-manured regimes. Principal coordinates analysis (PCoA) exhibited greater impacts of the addition of manure amendments than CFs on fungal community distributions. Redundancy analysis showed that the dominant fungal phyla were positively correlated with soil pH, soil organic C (SOC), total N, and microbial biomass C, and the fungal community structure was strongly affected by SOC. Network analysis explored positive relationships between microorganisms and could increase their adaptability in relevant environments. In addition, the structural equation model (SEM) shows the relationship between microbial biomass, soil nutrients, and rice grain yield. The SEM showed that soil nutrient contents and their availability directly affect rice grain yield, while soil fungi indirectly affect grain yield through microbial biomass production and nutrient levels. Our results suggest that manure application combined with CFs altered soil biochemical traits and soil fungal community structure and counteracted some of the adverse effects of the synthetic fertilizer. Overall, the findings of this research suggest that the integrated application of CF and manure is a better approach for improving soil health and rice yield.

## Introduction

Soil microorganisms are the primary driving forces in several key ecosystem processes, including soil structure formation, nutrient cycling, organic substance decomposition, and other soil biochemical processes; thus, soil microorganisms may help in monitoring soil fertility and health (Xiang et al., 2020; Vidal et al., 2021). Soil fungi are one of the most common soil microorganisms and important decomposers in the soil environment because they metabolize and transform organic matter for plant uptake by secreting specific extracellular enzymes (Mei et al., 2022). Therefore, changes in the key species involved in nutrient cycling can also affect soil fertility (De Beeck et al., 2021). Microorganisms can respond rapidly to changing environmental conditions by altering community composition and diversity indices (Du et al., 2022). Thus, understanding the spatial variation and the mechanisms regarding belowground microbial communities is essential for maintaining biodiversity under the paddy ecosystem in today's farming system (Luo et al., 2020).

The paddy ecosystem, which includes the world's third-largest cropland area and the largest anthropogenic wetland, is critical for global food security and environmental sustainability (Yuan et al., 2018). In today's conventional agricultural systems, paddy soils are routinely fertilized to improve soil fertility and enhance rice yield (Weifeng et al., 2022). However, the impacts of agricultural management on the soil microbial community are complex and diverse; particularly, different fertilization strategies had more profound effects on soil physicochemical and biological properties, resulting in a change in the microbial community (Li et al., 2022). Therefore, understanding how soil fungal communities respond to various fertilization strategies may provide a method for programmatically enhancing soil chemical and biological quality and soil function.

In the current farming system, chemical fertilizers, particularly synthetic N fertilizer inputs, have greatly contributed to the improvement of crop yield (Iqbal et al., 2021; Zhang et al., 2021). However, the adverse effects of CFs gradually emerged due to their overuse, such as a decline in nutrient use efficiency, deterioration in soil quality, decreased soil microbial respiration rate and activity, biodiversity losses, and a decrease in crop yield (Li et al., 2021; Zhang et al., 2021). Regarding the impact of synthetic fertilizers on communities of soil fungi, prior studies have reported that synthetic N application can decrease soil fungal biomass diversity and change fungal populations (Gomiero et al., 2011). Furthermore, the use of synthetic fertilizers alters fungal community structure and decreases external mycelium arbuscular mycorrhizal of soil fungi, whereas the use of organic fertilizers increases it (Paungfoo-Lonhienne et al., 2015; Jin et al., 2022). Alternatively, organic manure fertilization (i.e., farmyard and green manure) improves soil structure and fertility by increasing soil nutrient availability and organic matter (Zhang et al., 2021; Ali et al., 2022). The application of organic amendments has been reported to maintain microbial diversity and sometimes increase microbial populations (Liu et al., 2021). Manure application can change a soil's physical and biochemical properties, improve soil enzyme activities, and decrease or eliminate harmful impacts of the CF-only overuse on soil health (Zhang et al., 2022; Iqbal et al., 2022a). Therefore, a change in the source of N fertilizer is urgently needed to reduce the adverse effects of synthetic N fertilization.

Soil organic carbon (SOC), which is the main constituent of soil organic matter, forms the basis of soil fertility and is considered an important indicator of soil quality and sustainable agriculture (Yuan et al., 2021). The active SOC fractions were the main source of energy for soil microbes that could not only regulate crop nutrient availability but also optimize soil physicochemical properties (Xing et al., 2019). Moreover, soil fungal communities are significantly related to the soil C content and fertilization could impact soil fungal communities by changing soil nutritional status and plant biomass and physiology (Ali et al., 2022). Thus, it is necessary to characterize the variations in structure and diversity of soil fungal community and their relationship with soil properties, mainly C, under different manure and chemical fertilization treatments before implementing novel fertilization strategies to improve ecosystem health and crop productivity. This study is based on continuous research in paddy fields under dual cropping systems with the following research objectives: (i) illustrate the influence of manure and CFs' application on rice grain yield, soil properties, and fungal community composition and diversity and (ii) investigate the effect of soil chemical properties on soil fungal communities and the relationships between them. This study aimed to develop a theoretical framework for scientific fertilization practices and sustainable rice production while minimizing the use of chemical fertilizers.

## Materials and Methods

### Site Details

A continuous field study at the rice research farm of Guangxi University, China, was started in 2019. This location has a sub-tropical monsoon climate with ~1,300 mm of annual rainfall and an average annual temperature of 24.6 °C. The soil is defined as Ultisols (United States Department of Agriculture soil classification) and is acidic (pH 5.97). A soil test showed that the TN was 1.66 g kg^−1^ and SOC was 18.74 g kg^−1^; details of additional soil nutrients are presented in Table 1.

**Table 1:** Soil and organic manure chemical properties prior to experimentation.

**Sample**	**pH**	**SOC** **(g kg^**−1**^)**	**TN (g kg^**−1**^)**	**TP** **(g kg^**−1**^)**	**AN (mg kg^**−1**^)**	**AP** **(mg kg^**−1**^)**	**AK (mg kg^**−1**^)**
Soil	5.97	18.74	1.66	0.85	158	27.83	26.73
Cattle manure	7.83	161	9.57	11.1	-	-	-
Poultry manure	7.99	145	12.98	9.35	-	-	-

*TN, total nitrogen; SOC, soil organic carbon; TP, total phosphorous; AN, available nitrogen; AP, available phosphorous; AK, available potassium*.

### Experimental Setup

A double rice-growing season study (the early season runs from March to July and the late-season runs from July to November) was designed in a randomized complete block design with six treatments and three replications. Organic fertilizers [cattle manure (CM) and poultry manure (PM)] and chemical fertilizers (CFs), such as urea, were used, and the treatment combinations were: no N fertilizer control (Neg-CF); 100% CF in the form of urea (Pos-CF); 60% CM + 40% CF (high-CM); 30% CM + 70% CF (low-CM); 60% PM + 40% CF (high-PM), and 30% PM + 70% CF (low-PM). Rice seeds were grown in plastic trays, and the same-sized 25-day-old seedlings were transferred to the rice fields. Except for Neg-CF, which received no N fertilizer amendments, the recommended dose of N–P–K at a rate of 150:75:150 (kg ha^−1^) was used for every treatment (Sapkota et al., 2020; Iqbal et al., 2021). The nutrient composition and quantity of organic manure for each regime and the fertilizer application time are listed in Table 2. All organic fertilizers, including CM and PM, were applied 20 days before seedling transplantation. Furthermore, CFs containing N and K were applied at three different growth stages, namely, 50% before transplanting, 30% during tillering, and 20% at the heading stage. However, all P fertilizers were delivered as a base dose 1 day before seedling transplantation. Normal rice farming practices were performed for all regimes, such as irrigation (uniform flooding water was continued from seedling to rice maturity), pesticides, and insecticide application.

**Table 2:** Nutrient content and amount of nutrient provided of each plot and application time.

**Treatment**	**N (kg ha^**−1**^)**	**Urea** **(kg ha^**−1**^)**	**CM, PM, (kg ha^**−1**^)**	**Basal fertilization** **(kg ha^**−1**^)**	**Tillering** **(kg ha^**−1**^)**	**Panicle initiation** **(kg ha^**−1**^)**
Neg-CF	0	0	0	P_2_O_2_: 397, KCl: 128	KCl: 128	Urea: 00
Pos-CF: 100% CF	150	322	0	Urea: 192, P_2_O_2_: 397, KCl: 128	Urea: 65, KCl: 128	Urea: 65
High-CM: 60% CM +40% CF	150	130	9,188	Urea: 0, CM: 9,188, P_2_O_2_: 397, KCl: 128	Urea: 65, KCl: 128	Urea: 65
Low-CM: 30% CM +70% CF	150	225	4,572	Urea: 94, CM: 4,572, P_2_O_2_: 397, KCl: 128	Urea: 65, KCl: 128	Urea: 65
High-PM: 60% PM + 40% CF	150	128	6,623	Urea: 0, PM: 6,623, P_2_O_2_: 397, KCl: 128	Urea: 65, KCl: 128	Urea: 65
Low-PM: 30% PM +70% CF	150	225	3,290	Urea: 94, PM: 3,290, P_2_O_2_: 397, KCl: 128	Urea: 65, KCl: 128	Urea: 65

*N, nitrogen; CF, chemical fertilizer; CM, cattle manure; PM, poultry manure; P_2_O_2_, superphosphate; KCl, potassium chloride*.

### Soil Sampling and Analysis

#### Soil Properties

Soil samples were collected at depths of 0–20 cm using a core sampler at five different positions and combined to make a composite sample for each plot during the late and early rice season in 2019 and 2020, respectively, after panicle fertilization. The composite samples were divided into two parts and frozen at −80°C for later DNA extraction and microbial biomass C and N measurement. The second part was air-dried and used to determine soil chemical traits.

Soil organic C (SOC) was measured by the K_2_Cr_2_O_7_-H_2_SO_4_ oxidation process followed by titration (Wang et al., 2003). For total N (TN) analysis, 200 mg of soil samples were treated using the salicylic acid–sulfuric acid–hydrogen peroxide method described by Ohyama et al. (1991). Total N was determined using the micro-Kjeldahl technique as recommended by Jackson (1956). In addition, soil pH and available N, P, and K, were assessed by the methods of Lu (2000).

#### Microbial Biomass C and N

A fumigation extraction technique was used to determine microbial biomass C (MBC), as described by Brookes et al. (1985), and microbial biomass N (MBN) according to the procedure of Vance et al. (1987). We took 10 g of soil from the composite soil samples and divided them into equal halves. In a vacuum desiccator, 25 ml of ethanol-free CHCL_3_ was put in a petri dish to disinfect the first half of the soil (5 g) for 24 h at room temperature (25 °C). The samples were placed in warm water at 80°C after fumigation to eliminate fumes and 20 ml of K_2_SO_4_ (0.5M) was then used to remove C and N from both the fumigated and non-fumigated soils. The filtrated samples were then processed on a TOC Analyzer (TNM1; Shimadzu) and subjected to Kjeldahl digestion to calculate total C (TC) and TN. The following equation was used to estimate MBC and MBN:


$$\begin{array}{l} MBC\;or\;MBN=\frac{TN\;or\;TCfu\;-TN\;or\;TCnfu}{kEN\;or\;kEC} \end{array}$$

where TNfu and TNnfu are the total N in fumigated and non-fumigated samples, and TCfu and TCnfu are the TC concentrations in fumigated and non-fumigated samples. A kEC coefficient of 0.45 was used to estimate MBC according to Jenkinson et al. (2004), and a kEN coefficient of 0.54 was used to estimate MBN according to the method of Joergensen and Mueller (1996).

#### Grain Yield

The rice crop was harvested from the whole rice plot and rice grain yields were weighed at maturity. The dry weight of the rice grain was calculated using an adjusted moisture content of 14% in rice grains.

### DNA Extraction, PCR Amplification, and High-Throughput Sequencing

According to the manufacturer's instructions, sample DNA was extracted from 0.5 g of fresh soil using FastDNA Spin Kit for Soil (MP Biomedicals, LLC., Solon, OH, USA). The DNA integrity and concentrations were tested through electrophoresis and a NanoDrop®ND 2000 UV vis spectrophotometer (NanoDrop Technologies, Wilmington, DE, USA), and stored at −20 °C. The ITS1 region was amplified using primer ITS 5 F (5′-GGAAGTAAAAGTCGTAACAAGG-3′) and primer ITS 2 R (5′-GCTGCGTTCTTCATCGATGC-3′) (White et al., 1990). PCR mixtures (20 μl) contained 10 μl × TransStart Top Green qPCR SuperMix, 1 mM of each primer, 10 ng of ten-fold diluted DNA template, and 7.0–8.6 μl of Milli-Q water. The PCR thermal cycling conditions were as follows: 5 min at 95°C for the initial denaturation; 40 cycles of 5 s at 95 °C and 30 s at 58°C; and the final step of 40 s at 72 °C. The reactions were followed by melting curve analysis with temperatures increasing from 50 to 99 °C. As previously described by Kim et al. (2020), standard curves were generated. Products were pooled in equal amounts. Library preparation and sequencing were performed using the Illumina MiSeq300 platform (Illumina, San Diego, CA, USA).

### Sequencing Analysis

The original data were processed using the Quantitative Insights into Microbial Ecology (QIIME 1.9.0; Caporaso et al., 2010). High-quality sequences were clustered into operational Taxonomic Units (OTUs) at a 97% similarity threshold by UCLUST (Edgar, 2010). All singleton OTUs and chimera were deleted. Chimeric sequences were identified and removed using the USEARCH tool (Edgar, 2010). Taxonomic classification was carried out on representative sequences from each OTUs using the RDF classifier (Cole et al., 2009). Rarefaction curves of the species richness were plotted against the number of sequences using the MicrobiomeAnalyst (Dhariwal et al., 2017).

### Bioinformatics and Statistical Analysis

We estimated fungal alpha diversity indices, such as the Chao1 richness estimator, Shannon diversity index, Simpson index, and ACE metric (abundance-based coverage estimator), using MOTHUR (Schloss et al., 2009). A Venn diagram was used to visualize the number of common and unique OTUs of the samples and determine the similarity and overlap of the number of OTUs between the samples. A Venn diagram was generated using the R package “VennDiagram” (Zaura et al., 2009). The network analysis was performed using the “psych” package (v 2.0.12) in R and visualized by Gephi software (v 0.9.2; Dalcin and Jackson, 2018). Patterns of sample dissimilarity were shown by principal coordinate analysis (PCoA; Marsh et al., 2013). Based on the weighted Fast UniFrac distances between the samples, coordinates were used to draw graphical outputs of PCoA. Analysis of variance (ANOVA) was conducted to determine differences in soil chemical traits, rice grain yield, fungal α-diversity, and the abundance of the various taxonomic level of microbe between samples using software Statistics 8.1 (Tallahassee, FL, USA). The obtained data were initially tested for normality. Before analysis, data in percentages were arcsine-converted to normalize the variables. Tukey's *post-hoc* test was conducted to compare multiple means for the variables where the effects of experimental factors were significant. In addition, redundancy analysis (RDA) was performed using the software package CANOCO5 (Microcomputer Power, Ithaca, USA) to determine the correlation between soil environmental factors and soil fungal community composition functional group.

### Sequences Accession Number

The raw data are available in the National Center for Biotechnology Information (NCBI) Sequence Read Archive (SRA) database under Accession No. PRJNA806508 and link: https://www.ncbi.nlm.nih.gov/sra/PRJNA806508.

## Results

### Changes in Soil Chemical Properties

The combined application of manure and chemical fertilizers significantly increased the chemical properties of the soil, such as pH, SOC, and TN compared with sole chemical N fertilization: (Pos-CF), as shown in Table 3. The fertilization effect was highest in all measured variables when manure input was high; however, no significant (*P* < 0.05) differences between cattle and poultry manure were observed. All organic manure-amendment treatments significantly increased the SOC, TN, and pH compared to sole CF application treatments (Pos-CF). The soil pH of Pos-CF (pH 5.92) and high CM and PM (6.30 and 6.27) indicated that manure application increased soil pH. While the addition of CFs (Pos-CF) resulted in a lower pH.

**Table 3:** Changes in soil biochemical characteristics and rice grain yield under combined manure and chemical fertilizer application in 2020.

**Treatment**	**pH**	**SOC (g kg^**−1**^)**	**TN (g kg^**−1**^)**	**MBC (mg kg^**−1**^)**	**MBN (mg kg^**−1**^)**	**GY (kg ha^**−1**^)**
Neg-CF	5.97d	15.87c	1.42d	176d	23.54e	3468c
Pos-CF	5.92d	16.58c	1.43c	250c	35.49d	5516b
High-CM	6.30a	25.77a	1.98a	381a	53.52ab	6459a
Low-CM	6.20c	22.78b	1.77b	362b	44.37c	6744a
High-PM	6.27ab	25.55a	1.96a	379a	55.08a	6694a
Low-PM	6.19c	22.62b	1.79b	345b	42.36c	6852a

*Neg-CF, no N fertilizer; Pos-CF, 100% chemical N fertilizer (CF); High-CM, 60% cattle manure (CM) + 40% (CF); Low-CM, 30% CF + 70% CF; High-PM, 60% poultry manure (PM) + 40% CF; Low-PM, 30%PM + 70% CF, SOC, soil organic carbon; TN, total nitrogen; MBC, microbial biomass carbon; and MBN, microbial biomass nitrogen. The mean comparison was made using Tukey tests for treatment mean in both years and the lettering was done based on the Tukey HSD test at 5% using a simple effect. Values followed by the same letters within the column are statistically the same at P ≤ 0.05*.

In addition, high CM treatment increased SOC and TN by 55.4 and 38.5%, respectively, compared to Pos-CF. However, high CM was statistically (*P* < 0.05) similar to high PM. Similarly, low manured input such as low CM- and low PM- treated plots increased the measured soil chemical traits, i.e., SOC by 37.3 and 36.4%, and TN by 23.7 and 25.1%, respectively, compared to Pos-CF, as shown in Table 3.

### Changes in Soil Microbial Biomass C and N

The combined application of manure and CFs significantly increased soil microbial biomass C (MBC) and microbial biomass N (MBN) (Table 3). The combined use of cattle manure or poultry manure with chemical fertilizers significantly increased the soil MBC and MBN, compared to sole CFs. High CM treatment increased soil MBN and MBC by 54 and 62%, respectively, relative to Pos-Con. However, high CM was statistically (*P* < 0.05) similar to high PM. Similarly, low-manured input treatments also increased the measured soil biochemical traits as compared to Pos-CF.

### Changes in Rice Grain Yield

The integrated use of manure and synthetic fertilizer significantly affected the rice yield (Table 3). Combined fertilization increased rice yield considerably as compared with Pos-CF. Low CM and PM treatments increased the grain yield by 19 and 21% compared to Pos-CF. Similarly, high CM and high PM also significantly enhanced rice grain yield by 17 and 21%, respectively, compared with Pos-CF.

### Fungal Community Size and Diversity

**Figure 1:**
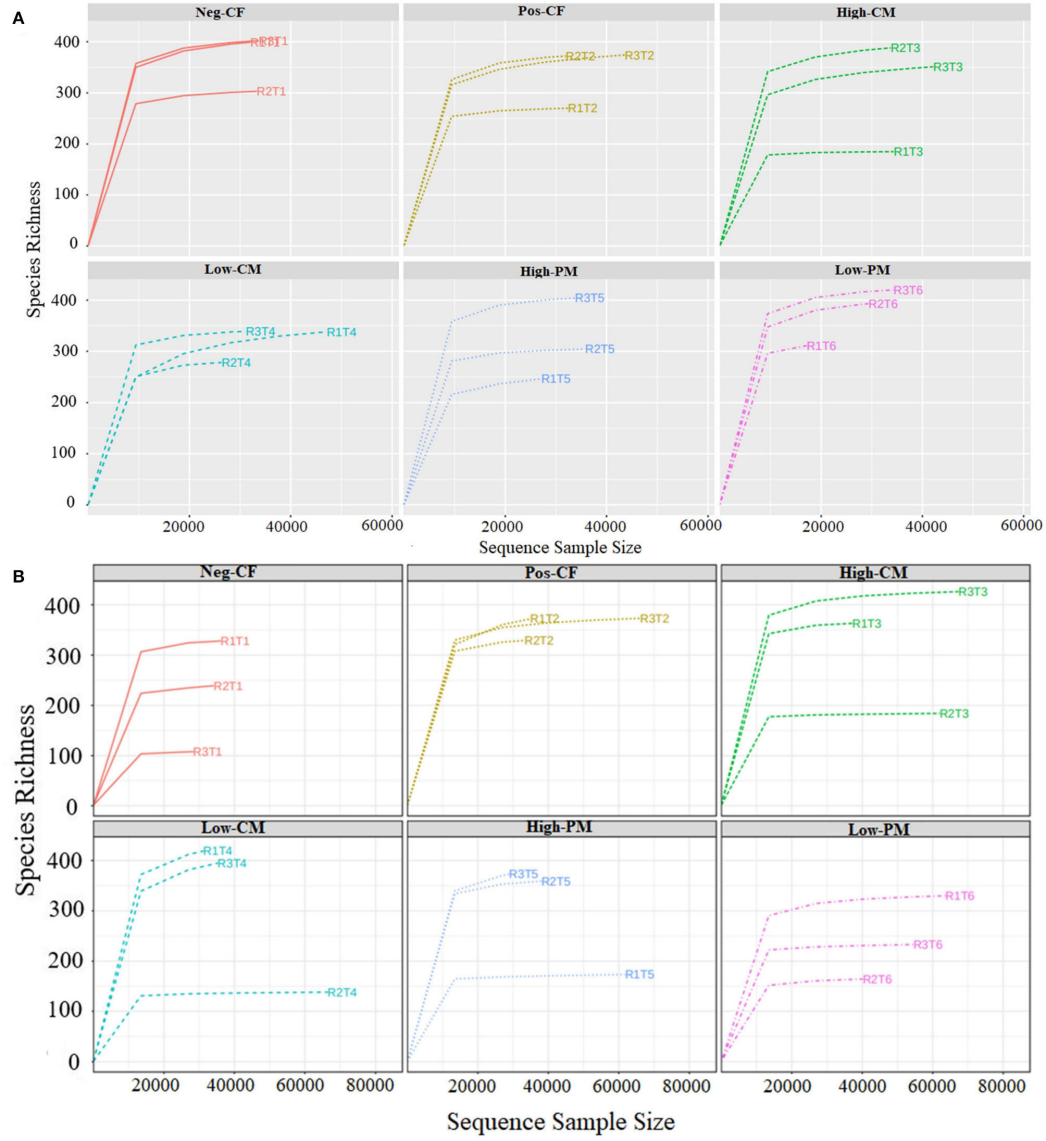
Rarefaction curves of ITS sequencing depth and number of species numbers in soil depth for year 2019 **(A)** and 2020 **(B)** under different combined organic and inorganic fertilizers application. Note: Neg-CF, no N fertilizer; Pos-CF, 100% chemical N fertilizer (CF); High-CM, 60% cattle manure (CM) + 40% (CF); Low-CM, 30% CF + 70% CF; High-PM, 60% poultry manure (PM) + 40% CF; Low-PM, 30%PM + 70% CF.

**Figure 2:**
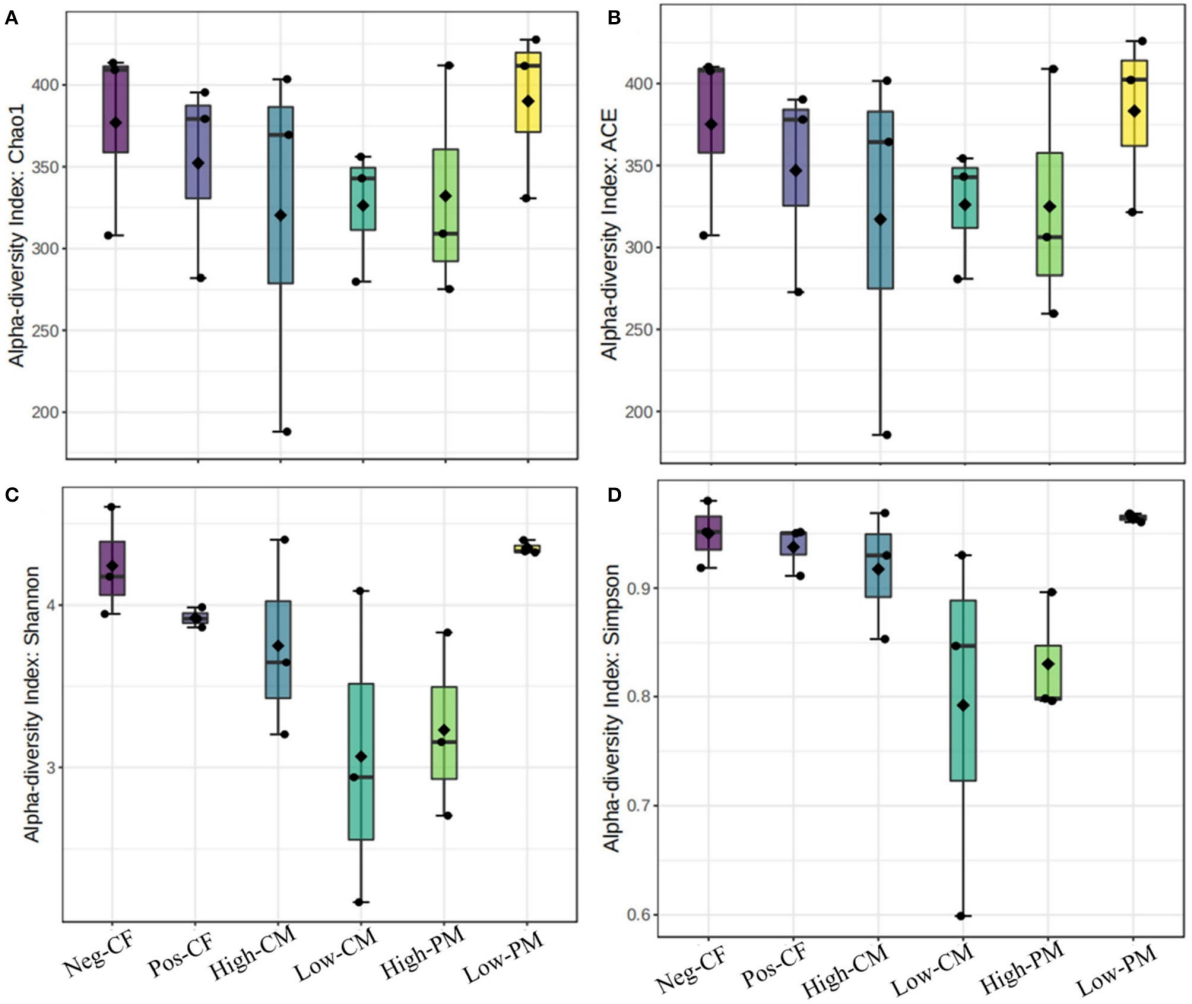
Comparison of the soil fungal communities based on qPCR-amplified ITS genes analysis. Box plots for fungal α-diversity [estimated Chao1, A.C.E., Shannon and Simpson indices in **(A–D)**, respectively] under different fertilization treatments in 2019. The ends of the whiskers represent minimum and maximum, the bottom and top of the box are the first and third quartiles, and the black dot inside the box is the median. Bars represent the standard error of the mean. Bars with different letters are significantly different at *P* < 0.05. Neg-CF, no N fertilizer; Pos-CF, 100% chemical fertilizer (CF); High-CM, 60% cattle manure (CM) + 40% (CF); Low-CM, 30% CF + 70% CF; High-PM, 60% poultry manure (PM) + 40% CF; Low-PM, 30%PM + 70% CF.

**Figure 3:**
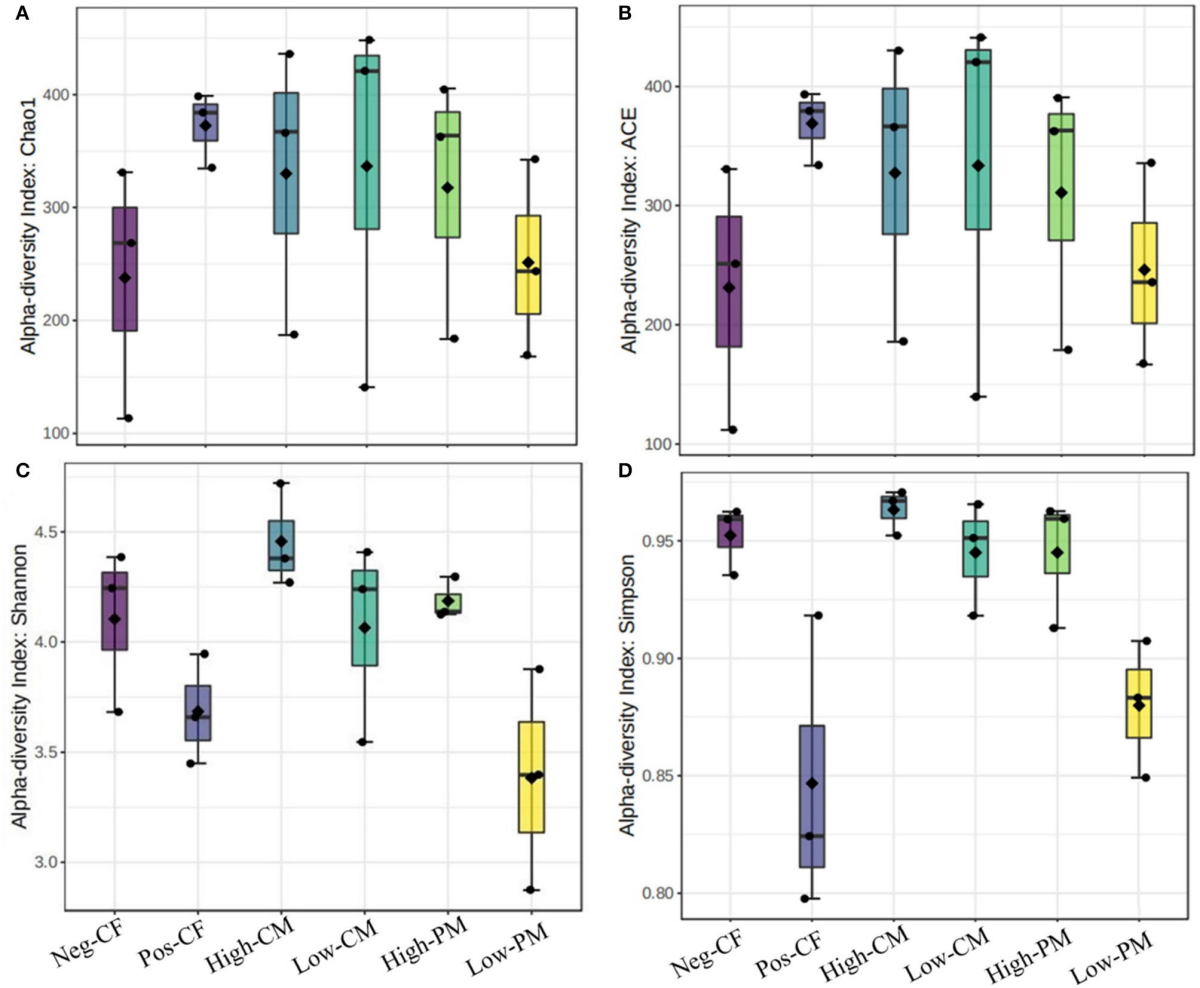
Comparison of the soil fungal communities based on qPCR-amplified ITS genes analysis. Box plots for fungal α-diversity [estimated Chao1, A.C.E., Shannon and Simpson indices in **(A–D)**, respectively] under different fertilization treatments in 2020. The ends of the whiskers represent minimum and maximum, the bottom and top of the box are the first and third quartiles, and the black dot inside the box is the median. Bars represent the standard error of the mean. Bars with different letters are significantly different at *P* < 0.05. Neg-CF, no N fertilizer; Pos-CF, 100% chemical fertilizer (CF); High-CM, 60% cattle manure (CM) + 40% (CF); Low-CM, 30% CF + 70% CF; High-PM, 60% poultry manure (PM) + 40% CF; Low-PM, 30%PM + 70% CF.

The fungal community size and diversity in samples were examined by qPCR targeting ITS rRNA. To determine rarefaction curves, richness, and diversity of bacteria, OTUs were identified at 97% of genetic similarity. The rarefaction curves exhibited that the sequencing effort was enough to describe most of the diversity in soil samples (Figures 1A,B). In this work, the integrated manure and mineral N fertilization significantly affected the soil bacterial community composition and diversity. The box plot based on the Chao1, ACE, Simpson, and Shannon indexes exhibited significant variations in soil fungal α-diversity detected in the fertilization treatments (Figures 2, 3). Co-fertilization of manure and chemical fertilizers resulted in the highest fungal diversity and abundance relative to Pos-CF. The number of community richness indices (ACE and Chao1) was higher in manured treatments than in sole CF application treatment. Furthermore, the combined manure and mineral fertilizer treatments resulted in higher community diversity indices (i.e., Shannon and Simpson) than sole CF-treated plots.

**Figure 4:**
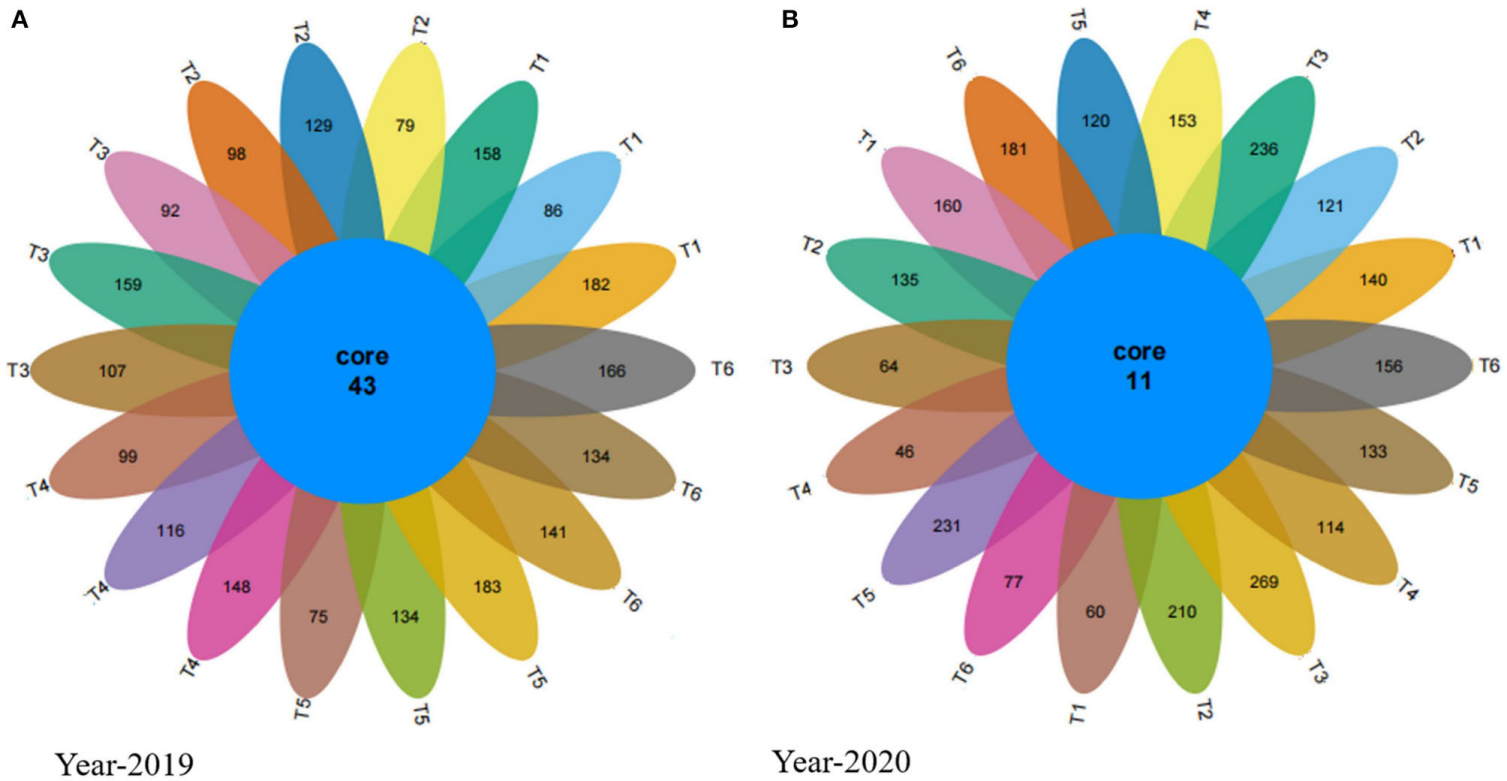
Venn diagram for the years 2019 **(A)** and 2020 **(B)** showing the fungal unique and operational units (OTUs) under different organic and inorganic fertilizer applications. T1-Neg-CF, no N fertilizer; T2-Pos-CF, 100% chemical fertilizer (CF); T3-High-CM, 60% cattle manure (CM) + 40% (CF); T4-Low-CM, 30% CF + 70% CF; T5-High-PM, 60% poultry manure (PM) + 40% CF; T6-Low-PM, 30%PM + 70% CF.

The Venn diagrams show that in 2019 the number of the unique average of the three replicates of OTUs in Neg-Con, Pos-Con, high-CM, low-CM, high-PM, and low-PM treatments was 142, 102, 119, 121,131, and 147, respectively, and the number of shared OTUs was 43 (Figures 4A,B). Similarly, in 2020, the number of unique OTUs was 120, 155, 190, 104, 161, and 138. The outcomes showed that OTUs in manure-treated plots were higher than in Pos-CF treatment. In this study, the increase of fungal OTUs numbers recorded under manured treatments compared to Pos-CF treatment, enhancement in OTUs number, and relative fungal community abundance in manured soils supported our previous hypothesis.

### Fungal Community Composition and Abundance

**Figure 5:**
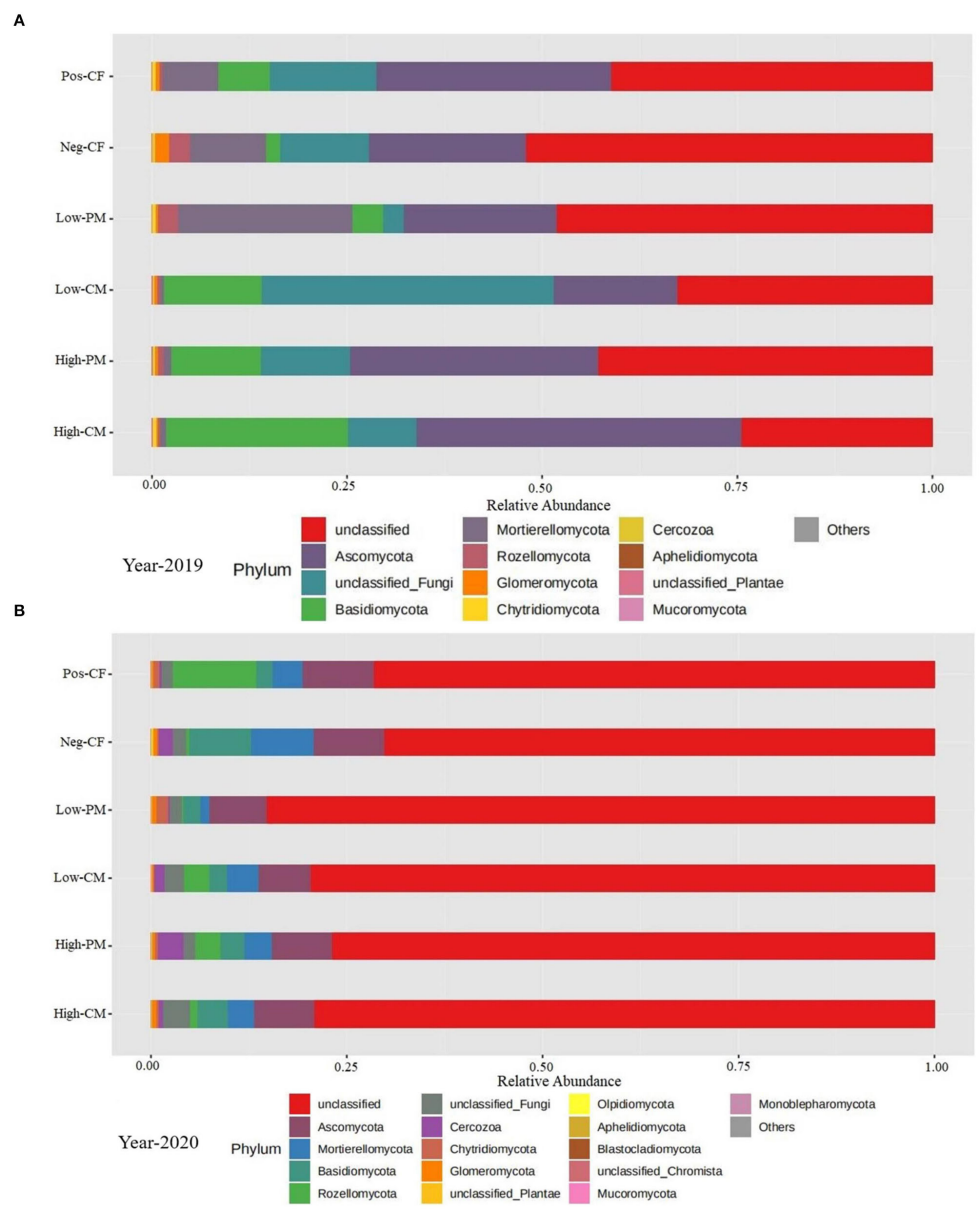
Relative abundance of soil fungi under different organic manure and inorganic fertilizer application at phylum level for year 2019 **(A)** and 2020 **(B)**. Note- Neg-CF, no N fertilizer; Pos-CF, 100% chemical fertilizer (CF); High-CM, 60% cattle manure (CM) + 40% (CF); Low-CM, 30% CF + 70% CF; High-PM, 60% poultry manure (PM) + 40% CF; Low-PM, 30%PM + 70% CF. Each strip represents the mean of three replicates.

The fungal community phylum composition of the six treatments is shown in Figures 5A,B. The soil analysis for fungal community composition under combined manure and chemical N fertilization indicated the presence of a total of 21 phyla, and the average relative abundance of 9 phyla exceeded 1%. The fungal species with the highest relative abundances were Basidiomycota, Ascomycota, Mortierellomycota, and Rozellomycota. Ascomycota was the dominant phylum in all treatments. The percentage was lowest in Pos-CF and highest for organic manured amendment treatments (i.e., high-CM, low-CM, high-PM, and low-PM). Ascomycota and Basidiomycota were the most abundant among these dominant phyla in the high-CM and high-PM treatments compared to the control (Pos-CF). Similarly, low CM and PM treatments also increased the abundance of Ascomycota and Basidiomycota compared with Pos-CF.

In addition, the six treatments showed remarkable changes in the abundance of the class levels of the fungal community (Supplementary Figure S1). The soil analysis for fungal community composition under combined fertilization showed the presence of a total of 23 classes, and the average relative abundance of 14 classes exceeded 1%. The top 5 dominant classes across all soil samples were Sordariomycetes, Dothidemycetes, Mortierellomycetes, Pezizomycetes, and Agaricomycetes. Sordariomycetes was the dominant class in all regimes, with the percentage lowest in Pos-CF and highest for manured amendment treatments.

### Network Structure of the Fungal Community

**Figure 6:**
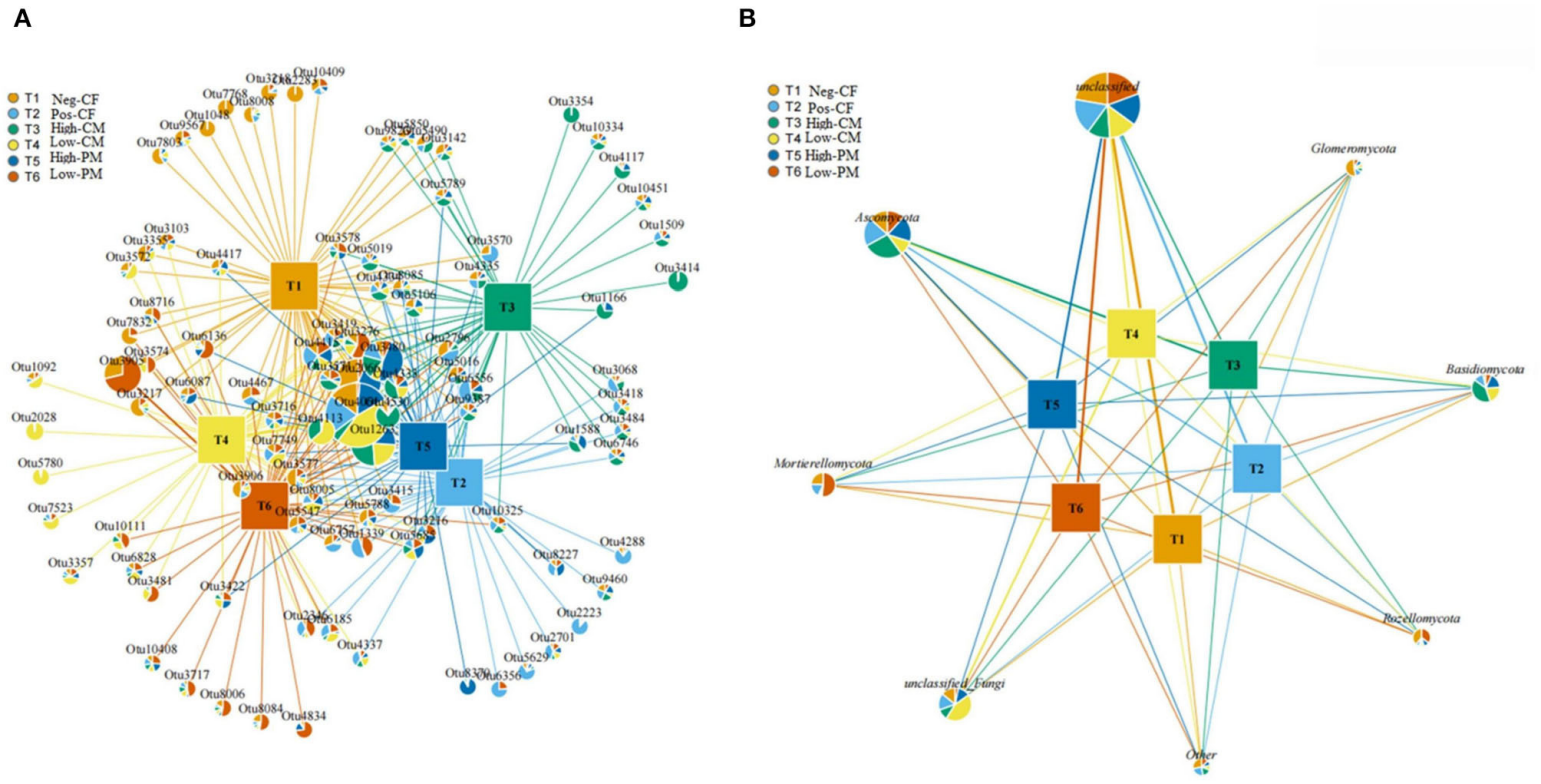
Fungal network diagram of the most abundant OTUs in soil samples **(A)** and diagram **(B)** shows the network analysis of OTUs at phylum level under different fertilization treatments.

An association network of the soil fungal community was constructed with the most abundant OTUs in the samples, comprising ~66.8% of the total sequences from the soil samples. The fungal community association network (Figures 6A,B) and Supplementary Tables 1, 2 showed the complete taxonomy for all the OTUs within the fungal networks. The resulting soil fungal network was analyzed with the more dominant phylotypes, representing 97.8% of the total sequences in the soil samples. The fungal association network consisted mainly of eight dominants associated with phyla (Figure 6B). One main network consisted of unclassified Fungi. The phylotypes belonging to Ascomycota (OTUs 224,694,730, and 830), Basidiomycota (OTUs 435, 818, and 1033), and Mortierellomycota (OTUs 2808, 3103, 2331, and 3245).

### Linkage Between the Soil Biochemical Properties and Microbial Structure

**Figure 7:**
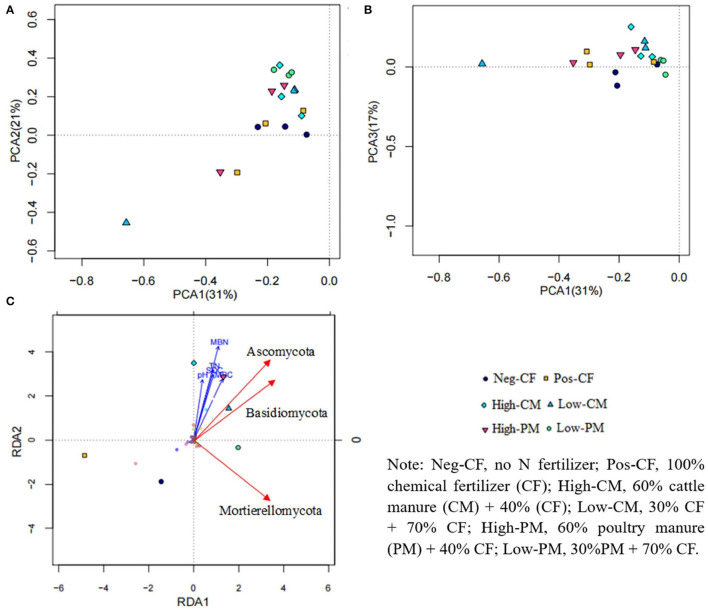
Principal coordinate analyses (PCoA) plot based on weighted UniFrac distance of soil fungal communities **(A,B)** sampled from each soil sample and results from RDA **(C)** to determine the relationship between soil fungal communities and soil properties.

Based on the weighted Fast UniFrac metrics, we conducted PCoA to examine the variation in soil fungal communities affected by continued manure and mineral fertilizer application. The first three principal coordinates (PCs) resulted in 31% (PCA1), 21% (PCA2), and 17% (PCA3) of the total variation in soil fungal communities (Figures 7A,B). The PCoA plot was exhibited in the upper part of PCA3 and was separated far from the manured amendment treatments. Similarly, PCA2 also showed that the Pos-CF and Neg-CF were separated from the manured treatments.

The RDA between different treatments, soil attributes, and soil fungal taxa exhibited that the soil biochemical traits contributed to the difference in soil microbial communities (Figure 7C). The analysis shows that the six treatments (Neg-Con, Pos-Con, high-CM, low-CM, high-PM, and low-PM) occurred in different quadrants, showing that the fertilization regimes had a substantial effect on the composition of soil microorganisms and soil properties (SOC, TN, MBC, and MBN). The RDA revealed that manure-treated treatments had a significant impact on soil fungal community composition than non-manure treatments. These findings show that applying manure amendments to rice fields has the greatest impact on soil microbial community composition and environmental properties.

Additionally, the correlation between soil biochemical traits and fungal community abundance at phylum and genus levels was performed by Person correlation analysis (Supplementary Figures S1, S2). Environmental variables (i.e., pH, TN, SOC, MBC, and MBN) were positively correlated with soil fungal community abundance. Similarly, the relationship of different fertilization treatments, especially manured amendments (i.e., high-CM, low-CM, high-PM, and low-PM) with the top thirteen phyla were positively associated (Figure 7). As a result, various organic manure and chemical fertilizers applications modified the pattern of soil fungal community composition by influencing soil health.

## Discussion

This study aimed to investigate the impact of continued manure and chemical fertilizer application on soil properties, microbial biomass production, and soil fungal community composition and diversity in paddy fields. The soil microbial community is the most crucial component of soil biology, responsible for enhancing soil fertility, health, and crop production (Liu et al., 2021; Shiau and Chang, 2022). This research showed that manure and chemical fertilizer application had a significant effect on soil biochemical traits and the abundance, composition, and diversity of fungal communities.

The combined application of manure and mineral fertilizers significantly increased soil biochemical indicators (such as microbial biomass C and N, pH, SOC, and TN) compared with CFs fertilization only. We found that manure biodegradation slowly released nutrients into the soil and showed that raising the rate of organic fertilizer (i.e., cattle or poultry manure) increased the soil's qualitative characteristics (Table 3). In this study, chemical fertilizer application reduced soil pH level, while combined manure and chemical N amendments increased the pH level significantly. The decline in soil pH level maybe because of the overuse of chemical N fertilizer forms H^+^ through nitrification in the soil (Yang et al., 2018). Moreover, the lower soil pH obtained by the CFs application compared to the manure-amended plots was because of the acidic nature of CFs, which could probably contribute to the less pH (Wang J. et al., 2021).

In this study, the manure and chemical fertilizer considerably enhanced the SOC and TN status in the paddy soil, which agrees with Han et al. (2021). The SOC at any specific position strongly relies on the annual organic turnover (i.e., plant root exudates, root and shoot stubbles, and their recycling) (Adekiya et al., 2020). The significant enhancement of SOC could be associated with the substantial effects of organic manure fertilization because the soil C change rate is affected by both direct C inputs from organic fertilizer and indirect C inputs from increased crop biomass returning to the soil (Iqbal et al., 2021, 2022b), such as crop and root residues. A previous study stated that the continuous application of organic fertilizers (organic manure) increased the SOC content and accumulation rates by enhancing crop yield organic matter returning from stubbles and roots (Yuan et al., 2021). Similarly, Iqbal et al. (2022b), reported that organic fertilizer significantly improved SOC in the top layer.

In addition, soil TN content was considerably increased under manured plots, as shown in Table 3. This is primarily due to the addition of manure, which has a significant effect on the soil TN, P, and K. This increment in N might also be allied with incorporating organic fertilizer remains, which directly added nutrients into the soil after decomposition (Zhang et al., 2021). Another possible reason for the increase in soil nutrients observed in this study is that organic fertilizers absorbed more leachate created throughout the process, resulting in increased water holding capacity, less nutrient leaching, and subsequently increased available nitrogen (Adekiya et al., 2020). Moreover, the increase in SOC content and accumulation significantly contributed to the increased N, P, and K contents in the current study. A previous study found that increasing C input enhanced nutrient availability by improving the physical and chemical characteristics of the soil, such as bulk density, aeration, and pH (Ullah et al., 2021).

Organic fertilizer, in conjunction with synthetic fertilizer, significantly increased soil microbial biomass C and N (Table 3). Increases in microbial biomass may have happened because manure enhanced the biogeochemical properties of the soil in the present study, leading to improved absorption of inorganic N by the plant (Iqbal et al., 2021; Ullah et al., 2021). Another reason is that organic amendments may have boosted soil nutrient richness and crop biomass accumulation, resulting in enhanced crop residues (Chen et al., 2021; Ali et al., 2022). In addition, organic fertilizers additions, such as high CM or PM and low CM or PM, significantly increased soil MBC and MBN in this study compared to sole CF application (Pos-CF). This discrepancy might be because the organic fertilizer carried a large amount of C that could drive microbial growth and activity (Ji et al., 2021; Zhang et al., 2021).

Co-incorporation of organic and chemical significantly improved rice grain yield compared to CF-only application in the current study. The increases in rice yield could be attributed to improved soil quality and fertility under manure fertilization, which ultimately enhanced rice growth and biomass accretion by providing enough nutrients during the growing period. After 2 years of application of organic fertilizers, the soil nutrients content in manured plots, particularly SOC and TN, were significantly higher than in Pos-CF treated plots. The improvements in grain yield could be due to the enhanced soil fertility and functionality under manured treatments, which ultimately increased crop growth, biomass accumulation, and yield by providing sufficient nutrients throughout the crop growing period (Iqbal et al., 2022b). Moreover, Ali et al. (2022) reported that changes in crop yield are strongly allied with soil biogeochemical properties and microbial biomass production. Iqbal et al. (2022b) reported that when the amount of soil organic C or soil organic matter increases, grain yield production increases consistently; this finding indicates that increasing the rate of organic matter in soils might be an essential factor in achieving higher rice yields.

Microbes are an important indicator of soil quality (van Bruggen et al., 2015). The richness and biodiversity of the soil microbial population are crucial to soil integrity, functionality, and sustainability; however, they are frequently reduced by conventional farming practices, such as overuse of chemical fertilizer and different tillage practices (Luo et al., 2020; Xiang et al., 2020). The fertilizers extensively affected the fungal community composition and structure in the current work, and the fungal communities were directly affected by organic amendments. Co-application of manure and mineral fertilizer significantly (*P* < 0.05) increased the number of sequences and the species richness (i.e., ACE, Shannon, and Chao 1 index values) of the fungi related to sole chemical fertilizer application (Pos-CF) (Figures 2, 3). According to Weifeng et al. (2022), organic manure applications include a wider range of substrates for microbe activities than synthetic fertilizers. They also directly introduce microorganisms found in manure into the soil.

The fungal abundance was positively correlated with soil organic carbon content (Figure 7C) demonstrating that organic fertilizer application increased fungal substrates to improve soil fungal abundance, which might, in turn, increase fungal function in nutrient cycling (Chen et al., 2021; Hou et al., 2022). Prior studies have reported that soil pH determined microbial diversity (Ali et al., 2020, 2021; Iqbal et al., 2022c). This study also found that soil fungal diversity showed a significant positive correlation with soil pH (Figure 7C), as the sole application of chemical fertilizers decreased soil pH, resulting in lower belowground fungal diversity (Figure 5). Soil acidification may increase environment filtering by niche selection, breaking the balance of the fungal taxa in the original habitat, thus leading to the extinction of certain taxa (Chen et al., 2020).

In this study, contrasting patterns of fungal community composition were observed following different fertilization treatments. Previous studies also reported the differential responses of the soil fungal community on the application of chemical and organic fertilizers (Xiang et al., 2020). Our results exhibited that the fungal community composition significantly (*P* < 0.05) increased by manure amendment-fertilization (Figures 5A,B). This was likely attributed to the availability of labile organic substrate and increased root exudates in the manured amendments soils (Li et al., 2020). Moreover, the fungal community structure in mineral fertilizers treatment and non-synthetic fertilizer treatment was similar; however, it changed following manure-amendment treatments. This is probably because the short-term input of mineral fertilizers did not considerably affect the soil pH. Soil pH is the most critical factor shaping the microbial community (Ramotowski and Shi, 2022). Organic amendments could enhance soil interspace and resource supply for microbes' growth (Li et al., 2020; Sun et al., 2020), thereby prompting microbe societies.

Additionally, a correlation heatmap revealed the associations between various fertilization regimes and the relative abundance of specific top fungal phyla (Supplementary Figure S2), which positively correlated to each other. Thus, the change in fungal community patterns was more significantly associated with applying different fertilizers. Moreover, correlation analysis demonstrated that changes in the soil fungal community could be mainly attributed to organic carbon inputs from organic amendments and varied fertilizer applications. It is observed that the integrated organic and mineral N fertilization increased the microbial community composition, diversity, and structure by making the soil atmosphere more suitable for the reproduction and activity of the fungal population (Wang J. L. et al., 2021; Sun et al., 2022).

Prior studies reported that soil pH was not only strongly correlated to microbial community and diversity (Lei et al., 2018) but also had a highly significant relationship with relative fungal abundance; thus, pH was thought to be a significant factor in determining the distribution of soil microbial community (Oladele et al., 2019). Similarly, RDA reported that soil pH influences the structure, diversity, and abundance of fungal communities in this study. Moreover, the fungal community abundance also showed strong relationships with SOC, TN, and microbial biomass C and N (Figure 7C). These findings indicated that nutrient element levels were the primary edaphic parameters impacting the composition of fungal communities. These results confirm that microbial community structure is usually most strongly allied with soil organic matter features, such as soil C, nutrient types, and soil fertility, because soil C and N are the primary energy foundations and component materials for fungi, and they influence the spread of soil fungi by regulating their metabolism (Op De Beeck et al., 2015).

Present information about soil microorganisms is primarily associated with prokaryotes, and the contents of the fungal communities of different fertilization soils are unclear. Research and understanding the microbial communities are of supreme importance since fungi comprise the main portion of biodiversity and biomass and play critical roles in preserving soil health that influences soil ecosystem functions. The network diagram illustrates positive and negative interactions among phylotypes and establishes co-occurrence patterns among taxonomic lineages under various fertilizers treated soils. Ascomycota, Basidiomycota, and Mortierellomycota were the dominant fungal phyla in different fertilized soils (Figure 6). These phyla contain essential genes that encode cellulose breakdown enzymes, increase C conversion, and play important roles in nutrient cycling (Bastida et al., 2013). According to a previous study, the phyla Ascomycota and Basidiomycota are the primary degraders of cellulosic organic matter and also participate in the soil C transformation process (Bahadori et al., 2022). Moreover, as the most dominant class in the manured soil in this study, Sordariomycetes, Dothidemycetes, Agaricomycetes, and Pezizomycetes have been confirmed to be key decomposers, producing both enzymes and hydrogen peroxide, degrading complex plant compounds, i.e., lignin and cellulose (Kameshwar and Qin, 2016). In this study, all network members had positive connections with one another, demonstrating that fungi in the soil can enhance their adaptation to adverse environments by managing species interaction.

**Figure 8:**
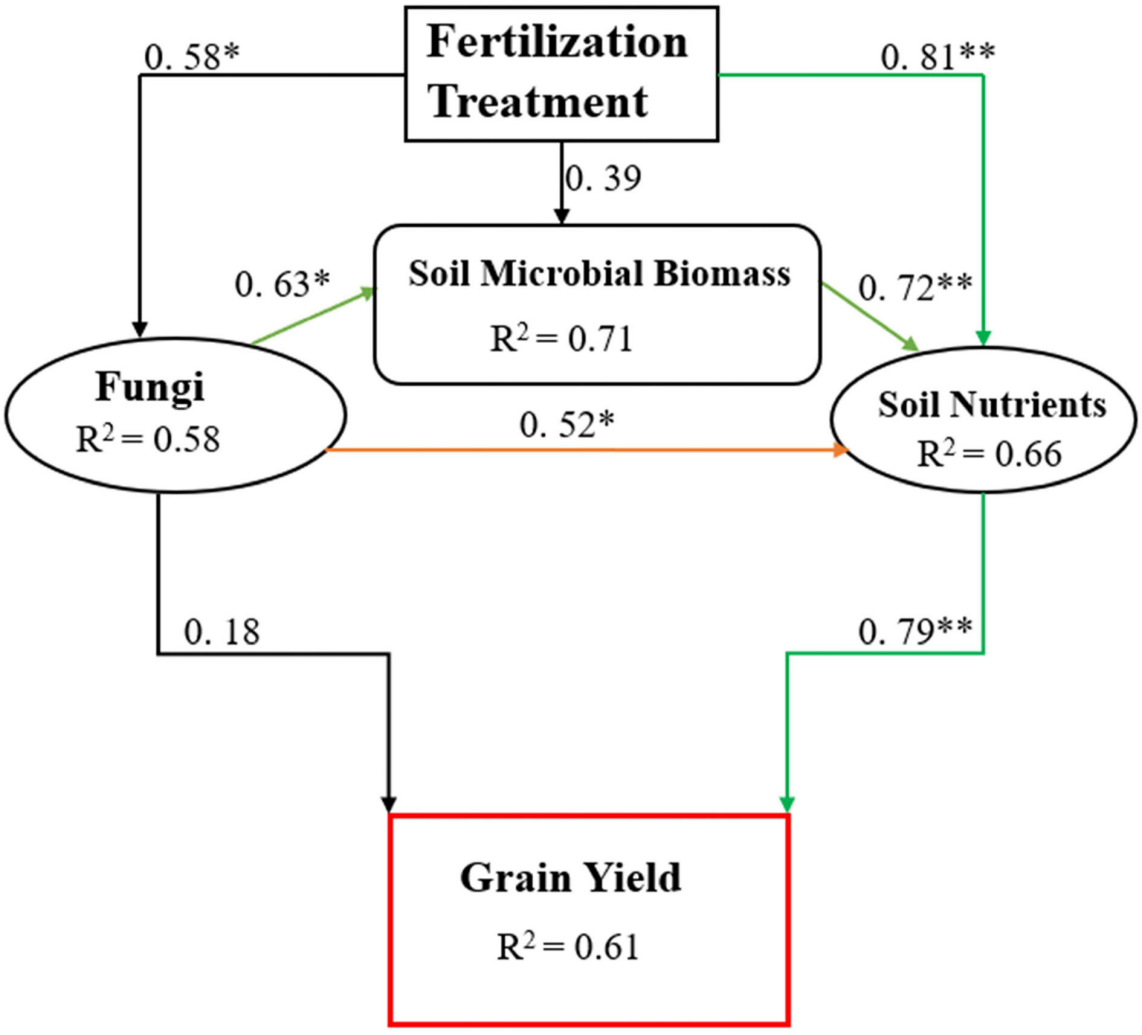
The value above the structural equation model line shows the path coefficient. The green lines represent the positive path coefficient and the black lines represent the non-significant path coefficient. The width of the arrow indicates the significance of the standard path coefficient (**P < 0.01 and *P < 0.05).

In this study, manure application combined with CFs improved the soil biochemical properties such as SOC, TN, MBC, MBN, and, ultimately, rice yield (Table 3). We found higher abundances of Ascomycota OTUs in the combined manure and CFs application treatments that might show better N and C cycling as they are important drivers of N and C cycling (Challacombe et al., 2019). Ascomycota and Basidiomycota are important fungal phyla involved in C cycling by degrading organic substances, and Ascomycota plays a dominant role in the degradation of organic matter in the rhizosphere, and changes in its abundance may affect soil fertility (Wang et al., 2020). The abundance of Basidiomycota is lower than Ascomycota because it is related to the degradation of plant liters in the forest with high lignin content, and their abundance may be lower in the cropping system (Francioli et al., 2016). Tayyab et al. (2019) also noticed a lower abundance of Basidiomycota in manure-amended soil, which supports the finding of this study. Other researchers also support the results of this study in terms of changes in fungal community structure with organic manure fertilization management (Ali et al., 2022). Thus, we employed a structural equation model (SEM) (Figure 8) to examine the relationship between microbial biomass, soil nutrients, and grain yield among treatments. The SEM showed that soil nutrient contents and availability affect rice grain yield while soil fungi indirectly affect grain yield through microbial biomass production and nutrient levels. We speculate from the above discussion and structural equation model that combined manure and CFs application improve soil microbial biomass and, ultimately, soil nutrients. Similarly, the combined manure and CFs treatments also change the soil fungal community and positively affect soil microbial biomass that enhances soil nutrients and thus increases rice production.

## Conclusion

In conclusion, the combined use of organic manure and inorganic fertilizers improves soil properties, the structure and diversity of fungal communities, and rice grain yield. The organic amendments significantly improved soil quality characteristics, including pH, SOC, TN, MBC, and MBN, as well as rice grain yield. Similarly, the combined use of CFs with cattle and poultry manure increased the composition of soil fungal communities compared to the use of CFs alone. The application of manure changed the structure of the fungal community in the soil and increased the relative abundance of Ascomycota, Basidiomycota, Mortierellomycota, and Rozellomycota phyla. Moreover, relative abundances varied dramatically at each taxonomic level, particularly between manured and non-manured plots. The substitution of animal manure regulated the relationship between soil fungi and soil properties, influencing the soil fungal community composition. PCoA and RDA further explored that the diversity of fungal communities was strongly related to the soil environmental variables (i.e., pH, SOC, TN, MBC, and MBN). The correlation analysis confirmed that soil C plays an important role in determining the structure and composition of fungal communities. This study found that applying organic manure and CFs (urea) significantly altered the structure of soil fungal communities, most likely by changing soil biochemical properties. Furthermore, the SEM highlights that the management of organic and inorganic fertilizers improves soil nutrient contents and availability directly, affecting rice grain yield, while the soil fungal community indirectly affects grain yield through microbial biomass production and nutrient levels. Consequently, combining manure and chemical fertilizers applications based on nutrient levels and proper fertilizer composition is the most effective approach for improving soil microbial community structure and fertility, ensuring rice grain yield and soil sustainability.

## Data Availability Statement

The original contributions presented in the study are publicly available. This data can be found here: BioProject, accession number PRJNA806508; https://www.ncbi.nlm.nih.gov/bioproject/PRJNA806508/.

## Author Contributions

AI and LJ: conceptualization and writing—original draft. AI, IA, and RK: methodology and formal analysis. AI, IA, and HL: investigation. LJ and SW: resources and supervision. AI, IA, HL, and PY: data curation. RK and LJ: writing review and editing. All authors contributed to the article and approved the submitted version.

## Funding

This study was fiscally funded by China's National Key Research and Development Project (2016YFD030050902).

## Conflict of Interest

The authors declare that the research was conducted in the absence of any commercial or financial relationships that could be construed as a potential conflict of interest.

## Publisher's Note

All claims expressed in this article are solely those of the authors and do not necessarily represent those of their affiliated organizations, or those of the publisher, the editors and the reviewers. Any product that may be evaluated in this article, or claim that may be made by its manufacturer, is not guaranteed or endorsed by the publisher.

## Abbreviations

OF: organic fertilizer
CF: chemical fertilizer
PM: poultry manure
CM: cattle manure
AN: available nitrogen
TN: total nitrogen
SOC: soil organic carbon
MBN: microbial biomass nitrogen
MBC: microbial biomass carbon
OTUs: operational taxonomic units.
