# Supplementary material for: Combined Application of Manure and Chemical Fertilizers Alters Soil Environmental Variables and Improves Soil Fungal Community Composition and Rice Grain Yield
Source: Front Microbiol. 2022 Jul 14;13:856355. doi: 10.3389/fmicb.2022.856355 (PMC9330912; doi:10.3389/fmicb.2022.856355)
Supplement: Supplementary file 1 [file Data_Sheet_1.docx]

**Supplementary material**


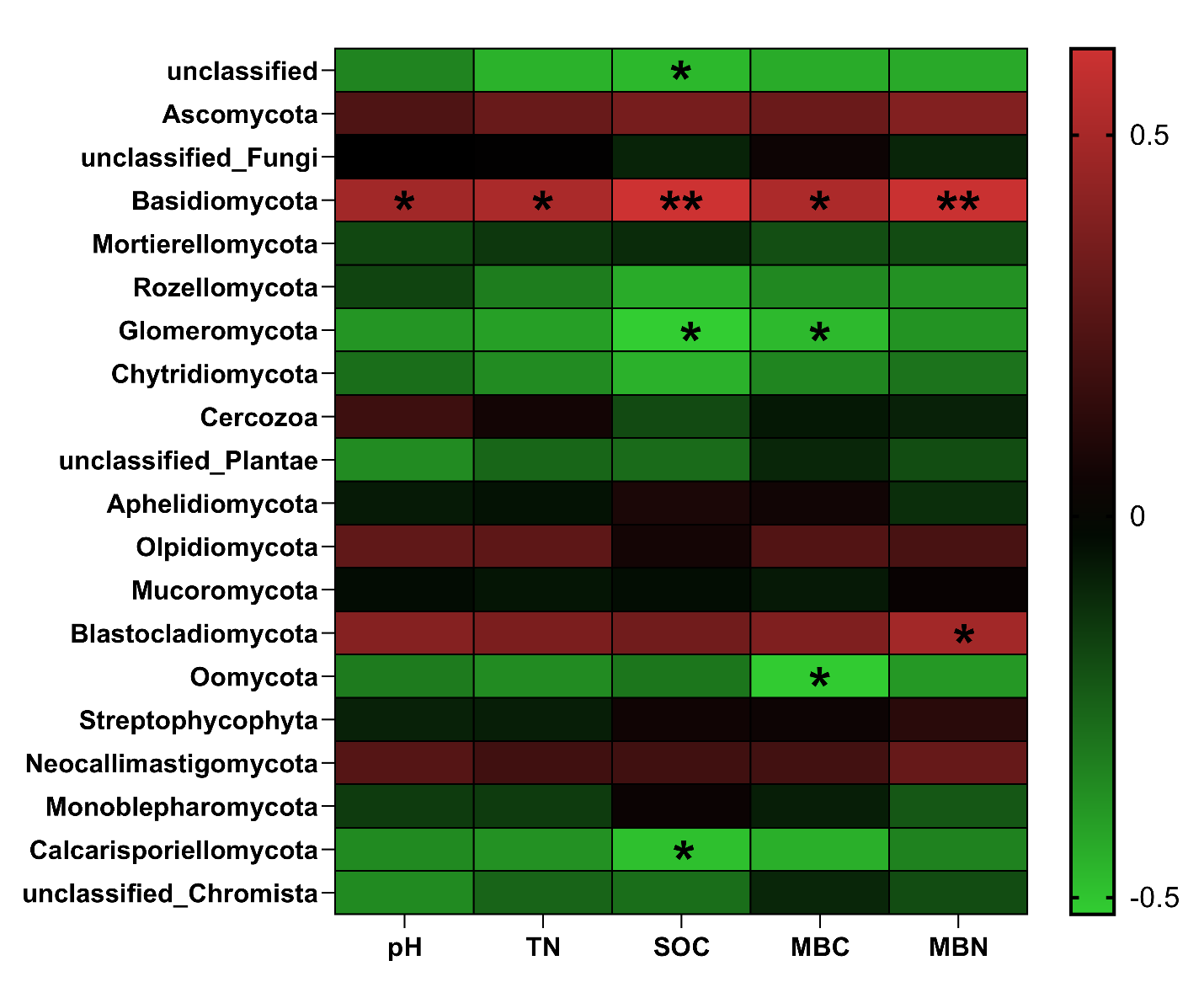
**Figure S1**. Correlation analyses between soil environmental variables and top phyla at the phylum level. The right side of the legend shows the color range and the corresponding correlation value. Note: *= Significance level at 0.05, and **=Significance level at 0.001.


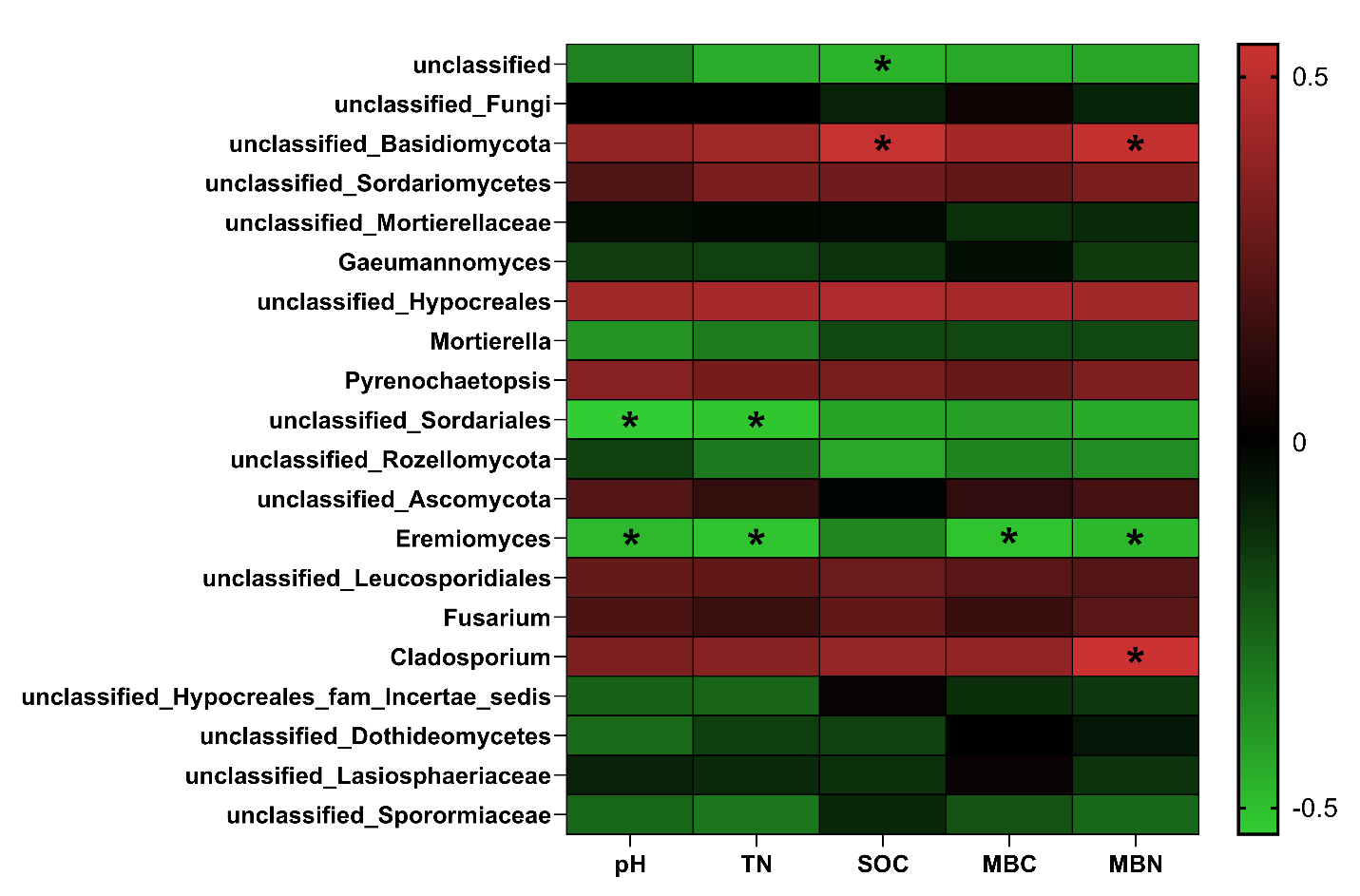


**Figure S2**. Correlation analyses between soil environmental variables and top phyla at the genus level. The right side of the legend shows the color range and the corresponding correlation value.

Note: *= Significance level at 0.05.


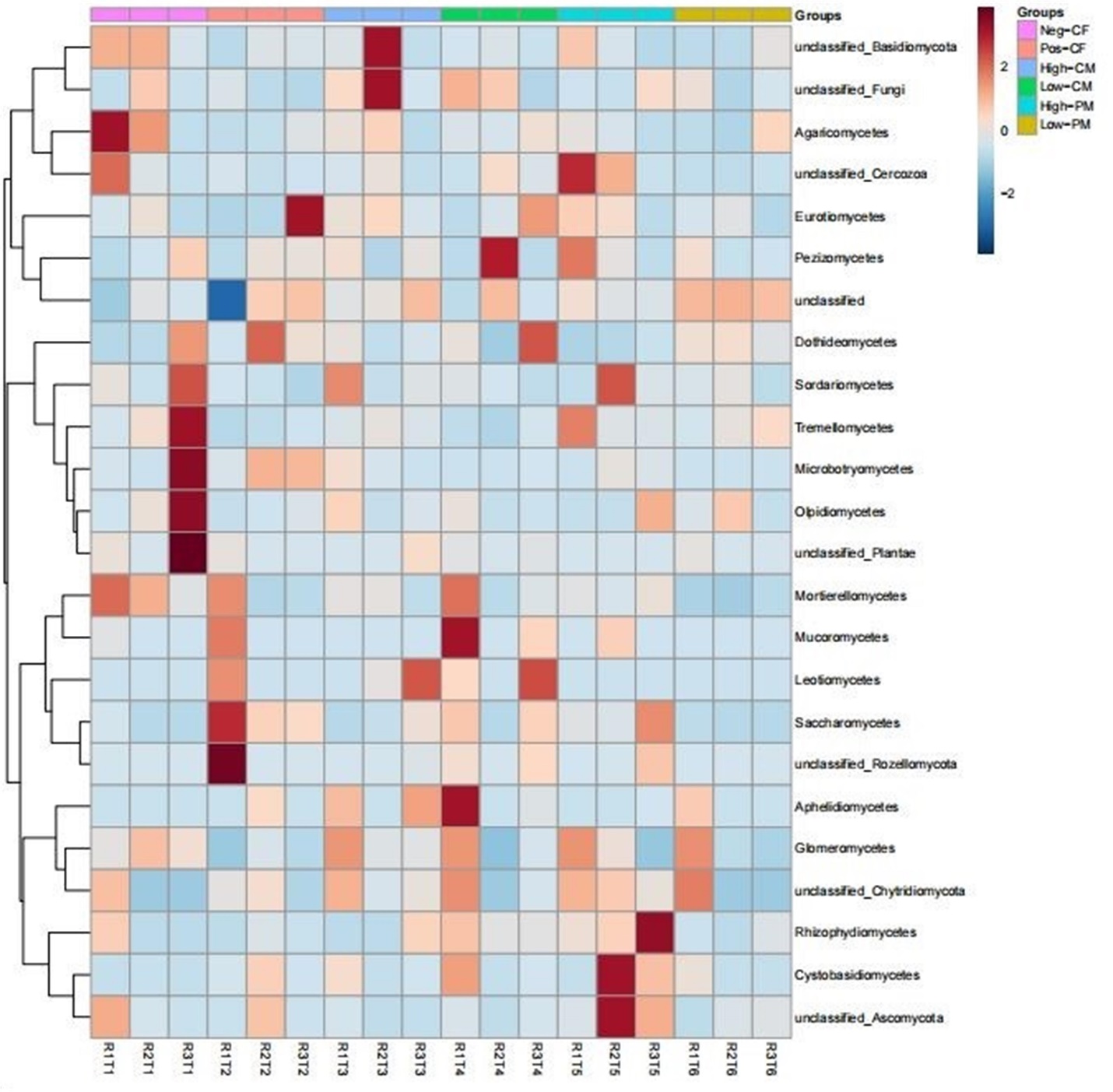


Figure S3. The heat map of the correlation between different fertilization treatments and the top thirteen phyla at class level. The right side of the legend shows the color range and the corresponding correlation value. Note- Neg-CF, no N fertilizer; Pos-CF, 100% chemical fertilizer (CF); High-CM, 60% cattle manure (CM) + 40% (CF); Low-CM, 30% CF + 70% CF; High-PM, 60% poultry manure (PM) + 40% CF; Low-PM, 30%PM + 70% CF.


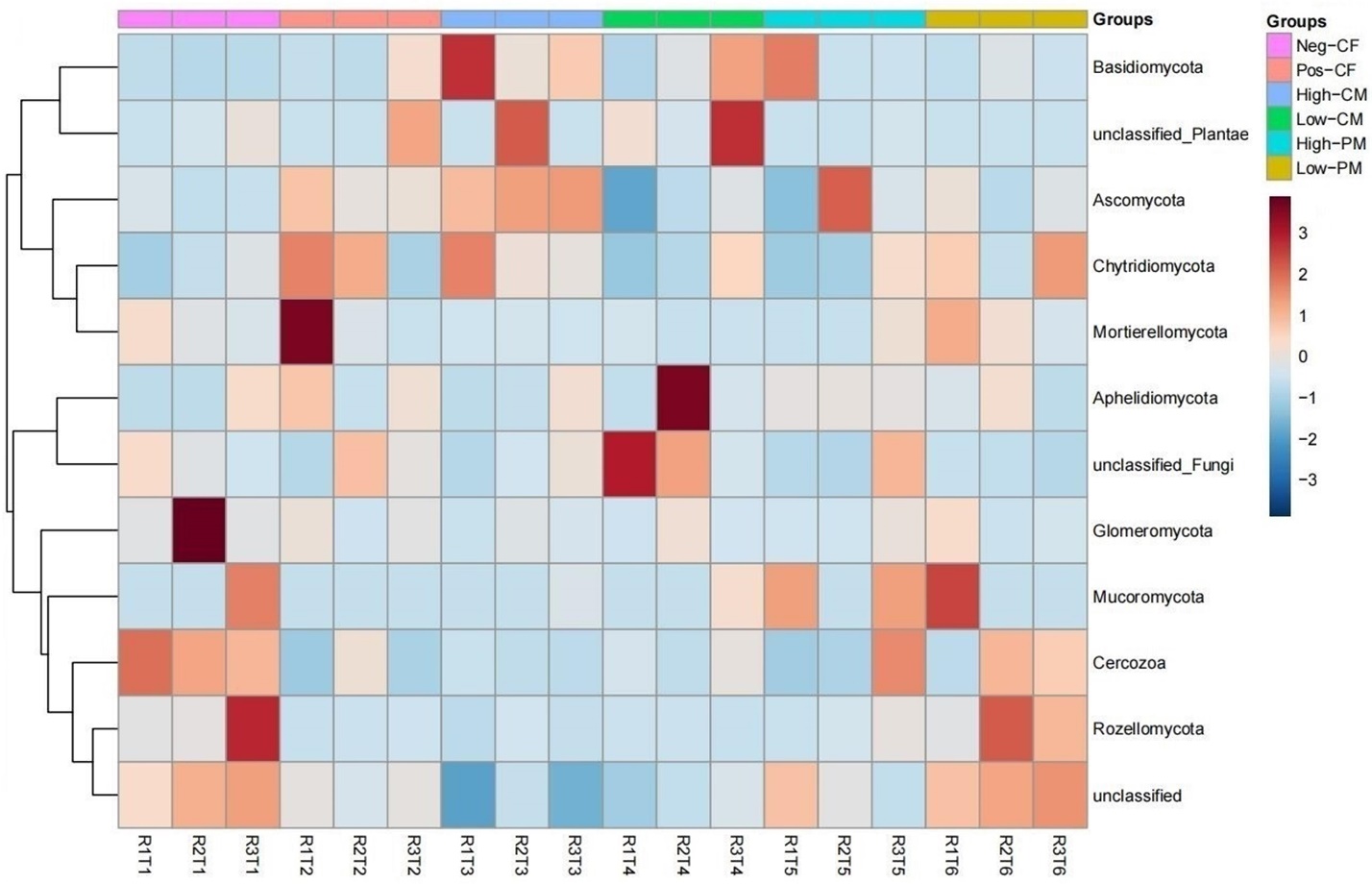


**Figure S3.** The heat map of the correlation between different fertilization treatments and the top thirteen phyla. The right side of the legend shows the color range and the corresponding correlation value. Note- Neg-CF, no N fertilizer; Pos-CF, 100% chemical fertilizer (CF); High-CM, 60% cattle manure (CM) + 40% (CF); Low-CM, 30% CF + 70% CF; High-PM, 60% poultry manure (PM) + 40% CF; Low-PM, 30%PM + 70% CF.
